# Mitogenomic analysis of *Palaeopsylla remota* and *Frontopsylla elata elata* with implications for the phylogeny of Siphonaptera

**DOI:** 10.3389/fvets.2026.1722404

**Published:** 2026-05-12

**Authors:** Shaobo Tang, Jun Wu, Mingna Duan, Rui Hou, Lanling Tian, Zongti Shao, Wei Gu, Xing Yang

**Affiliations:** 1 Integrated Laboratory of Pathogenic Biology, College of Preclinical Medicine, Dali University, Dali, China; 2 Dali Bai Autonomous Prefecture People's Hospital Ophthalmology, Dali, Yunnan, China; 3 Yunnan Institute of Endemic Disease Control, Dali, Yunnan, China; 4 Department of Infection, The First Affiliated Hospital of Dali University, Dali, China

**Keywords:** *Palaeopsylla remota*, *Frontopsylla elata elata*, Siphonaptera, mitochondrial genome, evolution, phylogenetic analysis

## Abstract

Fleas are among the most prevalent hematophagous ectoparasites found on mammals and have the ability to spread various pathogens. This study presents the first sequencing and analysis of the mitochondrial genomes of *Palaeopsylla remota* and *Frontopsylla elata elata*, alongside a comparative examination using mitochondrial genomic data from other flea species. Multiple aspects were investigated, including nucleotide composition, tRNA secondary structures, codon usage bias, nucleotide diversity, selective pressure, phylogenetic relationships, and divergence time estimation. Both species possess a typical mitochondrial genome comprising 37 genes and one control region, showing a pronounced A+T bias. Non-canonical G-U and U-U pairings were identified in some tRNA genes. ENC-plot and PR2-plot analyses indicated that natural selection is the main factor shaping codon usage bias in these two fleas. Within Siphonaptera, nucleotide diversity and selection pressure analyses revealed that the *cox1* gene exhibited the lowest values for both Pi and Ka/Ks. Phylogenetic reconstructions based on BI and ML methods using PCG123 and PCG12 datasets consistently supported the monophyly of the superfamily Ceratophylloidea and the family Pulicidae, whereas the families Ceratophyllidae, Leptopsyllidae, and Ctenophthalmidae displayed paraphyletic associations. Divergence time estimates suggest that the most recent common ancestor of extant fleas dates back to the Cretaceous period, with significant radiation events occurring after the K-Pg boundary. This study expands the mitochondrial genomic resources for fleas and offers new insights into their evolutionary history and phylogenetic relationships.

## 1 Introduction

Fleas are highly specialized, small, wingless insects characterized by their exceptional jumping ability and obligate hematophagous behavior. In their adult stage, they specifically parasitize the external surfaces of warm-blooded animals (1). Research indicates that approximately 94% of extant flea species are associated with mammals, particularly those inhabiting burrows or nests, while only about 6% have adapted to parasitizing birds (2). Furthermore, fleas are holometabolous insects, undergoing a complete metamorphosis that includes four distinct life stages: egg, larva (typically divided into three instars), pupa, and adult. Flea larvae predominantly inhabit the nests or burrows of their hosts, whereas adults are adapted to reside on the bodies or within the nest substrates of warm-blooded hosts (3). As external parasites that feed on blood, fleas can impact human health in multiple ways. Firstly, they can cause direct harm, such as skin injuries or allergic reactions in the host. During the feeding process, fleas inject saliva into the host’s skin to prevent blood coagulation, which may trigger a localized reaction, typically presenting as red spots and papules. In more severe cases, this may lead to allergic responses. Additionally, persistent itching may result in skin damage, secondary infection, and even suppuration at the bite site (3, 4). Moreover, fleas act as vectors for several significant human and zoonotic pathogens, including *Yersinia pestis* (plague), *Rickettsia typhi* (murine typhus), *R. felis* (flea-borne spotted fever), and *Coxiella burnetii* (Q fever) (5–7). Furthermore, as intermediate hosts, fleas can transmit several parasitic tapeworms to humans and other mammals, including *Dipylidium caninum* (causing dog tapeworm infection), *Hymenolepis diminuta* (rat tapeworm infection), and *H. nana* (dwarf tapeworm infection) (8–10). In the process of conducting epidemiological investigations on fleas by health regulatory authorities, it is crucial to accurately and rapidly identify flea species. Currently, the identification and recognition of flea species primarily depend on morphological characteristics, while relevant research in molecular biology remains relatively scarce. Morphological identification is not only constrained by environmental changes but also poses challenges in accurately distinguishing flea larvae, individuals with incomplete bodies, or those that have undergone evolutionary changes. Additionally, vector species exhibit morphological homogeneity during certain life stages. Collectively, these factors render the rapid and precise identification of flea species quite challenging (11).

The phylogenetic relationships and taxonomic classification within the order Siphonaptera have long been a focal issue in insect systematics. Traditional morphological studies support the monophyly of Siphonaptera. A 2014 phylogenomic study on insect evolutionary patterns and timing also supported the monophyly of both Siphonaptera and Mecoptera (12). However, a multigene-based phylogenetic analysis utilizing *18S rRNA*, *28S rRNA*, *cox2*, and *EF-1α* yielded a different conclusion: it suggested that Mecoptera is paraphyletic, with Siphonaptera originating from within Mecoptera and forming a sister group relationship with Boreidae. Additionally, Nannochoristidae was recovered as the sister group to the clade comprising Boreidae and Siphonaptera (13–16). In 2020, Tihelka et al. employed a dataset of 1,478 orthologous single-copy nuclear protein-coding genes (PCGs), and their analysis also placed Siphonaptera within Mecoptera, supporting a sister group relationship with extant Nannochoristidae (17). Notably, a 2022 study based on mitochondrial genomes reached a different conclusion, suggesting that Siphonaptera forms a sister group relationship with a clade comprising Diptera, Mecoptera, Megaloptera, and Neuroptera (18). These results indicate that the phylogenetic placement and taxonomic status of Siphonaptera remain highly controversial. Future efforts integrating higher-quality molecular data will be essential to accurately resolve the phylogenetic relationships within Siphonaptera, which will also provide a crucial theoretical foundation for the rapid and accurate identification of flea species.

Mitochondria are essential organelles involved in cellular energy metabolism and biosynthesis, participating in various fundamental physiological processes, including oxidative phosphorylation for ATP production, fatty acid oxidation, phospholipid biosynthesis, and the translation of specific proteins (19, 20). As semi-autonomous organelles, mitochondria possess their own genetic material, known as mitochondrial DNA (mtDNA) (21). Compared to nuclear genes, mitochondrial genomes are characterized by their smaller size, maternal inheritance, higher evolutionary rates, and greater conservation. Consequently, mitochondrial genomes have become the most widely used DNA barcoding tool for species identification, making them suitable for investigating phylogenetic relationships among species, population genetic differentiation, and evolutionary history (1, 22–25). However, the mitochondrial genomic data for Siphonaptera insects remains extremely limited. Current statistics indicate that fewer than 40 species of flea mitochondrial genomes have been sequenced globally. This data deficiency significantly hinders the advancement of molecular evolution and phylogenetic research on fleas. Therefore, expanding the mitochondrial genome database for fleas is crucial for fostering in-depth research in this area. This study represents the first analysis of the mitochondrial genomes of *Palaeopsylla remota* and *Frontopsylla elata elata*. It not only fills a significant gap in the mitochondrial genome data of fleas but also provides an important molecular basis for taxonomic, population genetic, and phylogenetic research on fleas.

## 2 Materials and methods

### 2.1 Specimen collection, morphological identification, and DNA extraction and sequencing

In this study, *P. remota* was collected from Lushui City, Nujiang Lisu Autonomous Prefecture, Yunnan Province, China (25°50′39.2″N, 98°51′25.1″E), in August 2024, and *F. elata elata* was collected from Jinghe County, Xinjiang Uygur Autonomous Region, China (44°36′3.3″N, 82°53′22.0″E), in July 2024. The Ethics Committee of Dali University granted approval for the specimen collection and experimental procedures utilized in this research (Application number: 2024-SL-280; Approval number: 2024-P2-280), which also obtained the necessary authorization from the pertinent local administrative authorities in Jinghe County, Xinjiang Uygur Autonomous Region, as well as Lushui City, Nujiang Prefecture, Yunnan. All specimens underwent immediate surface cleaning treatment following collection. Subsequently, professional taxonomists identified the species based on external morphological characteristics, adhering to the authoritative classification standards outlined in the “*Fauna Sinica insecta Siphonaptera*” (26). After completing the morphological identification, the individual specimens were preserved in EP tubes containing 95% ethanol and stored in a −20 °C ultra-low temperature refrigerator for cryopreservation, intended for subsequent molecular biological research. Genomic DNA from the two types of fleas was extracted in strict accordance with the manufacturer’s operational procedures using the TIANamp Genomic DNA Kit (Tianamp Biochemical DNA Kit, Beijing). Following the quality inspection of the final DNA samples, they were sent to Shanghai Paisenor Biotechnology Co., Ltd. for high-throughput sequencing. Sequencing was conducted on the Illumina NovaSeq 6,000 platform in PE150 mode. The raw data yielded 1.38 Gb (11,818,818 reads; GC: 34.61%; Q20: 95.79%; Q30: 90.05%) for *P. remota* and 0.796 Gb (7,598,124 reads; GC: 32.36%; Q20: 96.55%; Q30: 91.02%) for *F. elata elata*.

### 2.2 Mitochondrial genome assembly and annotation

Data quality control was conducted utilizing fastp^1^ to confirm the collection of high-quality data. The raw data obtained from sequencing typically contains adapter sequences, low-quality reads, and ambiguous bases, which can interfere with the accuracy of subsequent analyses. To ensure the quality of subsequent information analysis, it was essential to filter the raw sequencing reads, thereby generating high-quality sequences (clean data). After quality filtering, the clean data totaled 1.24 Gb (11,720,358 reads) for *P. remota* and 0.737 Gb (7,386,328 reads) for *F. elata elata*. Subsequently, the high-quality sequencing data were assembled with SPAdes v3.15.4 (27) and GetOrganelle v1.7.7.0^2^. Based on the sequencing depth of the assembled sequences, high-depth sequences were extracted and aligned against the NCBI nt database using BLASTn (BLAST v2.2.31+) to identify mitochondrial sequences from each assembly. Bandage v0.8.1^3^ was used to determine the connections and positional relationships among mitochondrial fragments. If gaps existed between fragments, sequencing reads were used to extend the assembly ends until a complete circular structure was formed. Finally, the assembly was polished using Pilon v1.18 to obtain the final mitochondrial genome sequences (28). A sequencing depth of ≥100 × is generally considered indicative of high assembly accuracy. In this study, the sequencing depths for both species exceeded 100×, ensuring high single-base accuracy for the mitochondrial genomes of *P. remota* and *F. elata elata* (Supplementary Figure S1). The assembled complete mitochondrial genome sequences were then submitted to the MITOS web server^4^ for functional annotation (29). The secondary structures of tRNA genes were predicted using tRNAscan-SE v.2.0 (30) and ARWEN v.1.2.3 (31).

### 2.3 Sequence analysis

The composition of the base pairs, including A + T and G + C content, was determined using DNAstar v6 (32). Additionally, the AT-Skew and GC-Skew were computed according to the formulas: AT−Skew=(A−T)/(A+T) and GC−Skew=(G−C)/(G+C) . The 13 PCGs were accurately extracted from the mitochondrial genome using PhyloSuite v1.2.3 software (33). Further analysis was conducted with DnaSP v6 software as follows: (1) The selection pressure was evaluated by calculating the nonsynonymous substitution rate (Ka), the synonymous substitution rate (Ks), and their ratio (Ka/Ks) for each gene; (2) The nucleotide diversity (Pi) of the 13 PCGs was analyzed using the sliding window method, with a window size of 100 bp and a step size of 25 bp.

Codon usage patterns in the 13 PCGs were assessed with CodonW 1.4.2. The analysis included calculations of relative synonymous codon usage (RSCU), effective number of codons (ENC), nucleotide composition at the third codon position (A3s, T3s, G3s, C3s), as well as GC content at the third position (GC3s). In order to explore the factors influencing codon usage bias, we created ENc-plot graphs, with ENC values represented on the Y-axis and GC3s on the X-axis. Theoretical curve fitting utilized the standard curve equation: ${\mathrm{ENC}}_{\text{expected}}=2+\mathrm{GC}3s+29/(\mathrm{GC}3s^{2}+{\left(1-\mathrm{GC}3s\right)}^{2})$ . When gene points are situated near the theoretical curve, it suggests that codon usage is predominantly influenced by mutation bias. Conversely, if genes are positioned below the theoretical curve and at a considerable distance from it, this indicates the potential impact of natural selection or other factors (34). The Parity Rule 2 (PR2) analysis strategy was employed, calculating the ratios A3/(A3 + T3) and G3/(G3 + C3) at the third position of the genes to construct PR2-plot graphs with vertical and horizontal coordinates, respectively. Theoretically, in the absence of deviation from natural selection and mutational pressure, A should equal T and G should equal C, with this point serving as the coordinate center. When gene points deviate from this center, their vector direction and distance can reflect the extent and direction of bias: if A ≠ T or G ≠ C, it indicates that codon usage preference is influenced by natural selection and mutational pressure (35, 36). Finally, R Studio was utilized to conduct the visual analysis of the RSCU plot, ENC-plot, and PR2-plot.

### 2.4 Phylogenetic analysis

Mitochondrial genomes of flea species available on NCBI were used for phylogenetic reconstruction, using *Boreus elegans* as the outgroup. This analysis was based on two data matrices: (I) full codon tandem nucleotide sequences of 13 PCGs (PCG123); and (II) the first and second codon tandem nucleotide sequences (PCG12) of the same genes. Both datasets were used to reconstruct phylogenetic trees under Maximum Likelihood (ML) and Bayesian Inference (BI) frameworks. Sequence alignment for the PCG123 and PCG12 datasets was performed using MAFFT software (37), and the optimal nucleotide substitution model was determined via ModelFinder. ML phylogenetic trees were constructed with IQ-TREE v.1.6.12 (38) setting the number of bootstrap replicates to 1,000. Meanwhile, BI phylogenetic trees were computed by MrBayes v.3.2.7 (39), where the Bayesian analysis was run for 2,000,000 generations, and a sample was taken every 1,000 generations. We used four independent MCMC chains and burned-in one-fourth of the generations sampled. Finally, the online tool iTOL^5^ was utilized to visualize and enhance the phylogenetic trees.

### 2.5 Divergence time estimation

We estimated divergence times using MCMCTree v4.10.7. The analysis was performed on a well-supported phylogenetic tree topology obtained through Bayesian inference, which was constructed based on 13 PCGs. Given the general scarcity of definitive Siphonaptera fossils, we selected only unequivocally identified flea specimens preserved in amber as calibration points to ensure dating reliability. Such confirmed Siphonaptera fossils provide reliable empirical evidence for the objective existence of specific evolutionary lineages during geological time periods. (I) *Pulex*: This fossil is morphologically consistent with the extant genus *Pulex*. Although it is dated to the Miocene, the geological age of Dominican amber is contentious, with estimates ranging from 20–15 to 45–30 Mya. To incorporate this uncertainty, we conservatively assigned an age range of 35–15 Mya (40). (II) *Palaeopsylla*: Fossils of the genus *Palaeopsylla* have been recovered from Baltic amber, with its geological age widely accepted as 40–35 Mya (41–43). We employed the independent rates model (clock = 2) as the molecular clock and selected the JC69 base substitution model (model = 0). The prior Settings are as follows: BDparas = 1 1 0.1, rgene_gamma = 2 20 1, and sigma2_gamma = 1 10 1. To ensure reliability of the results, we ran two independent Markov chain Monte Carlo (MCMC) chains, each with 1,000,000 generations, discarding the first 200,000 generations as burn-in. Convergence was assessed using Tracer v.1.7.2, confirming that all parameters had effective sample sizes (ESS) greater than 200. The resulting divergence time tree was subsequently visualized and refined using the tvBOT tool.

## 3 Results

### 3.1 General structure of the mitochondrial genomes of *Palaeopsylla remota* and *Frontopsylla elata elata*

**Figure 1:**
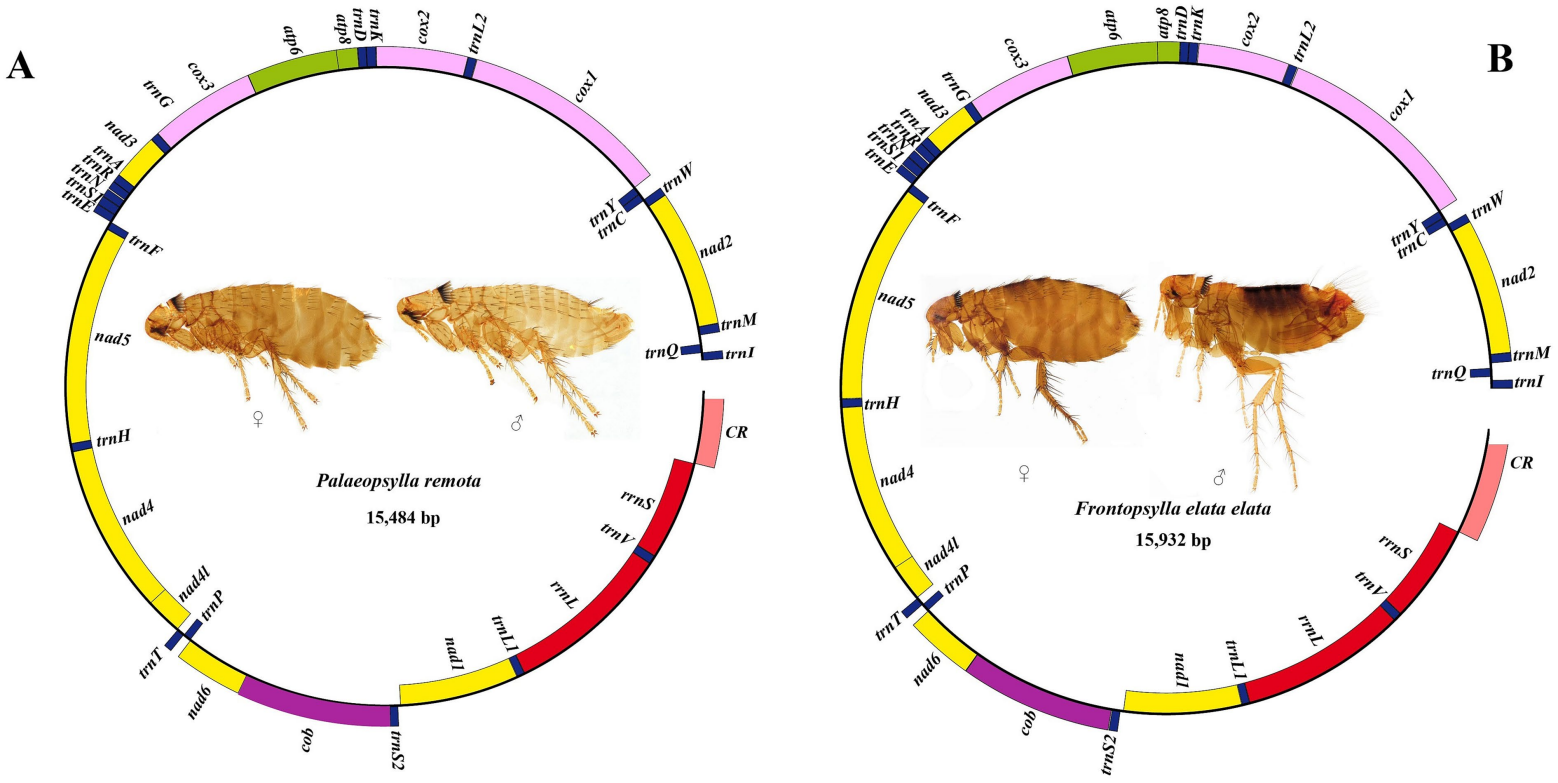
Mitochondrial genome organization of *Palaeopsylla remota* **(A)** and *Frontopsylla elata elata* **(B)**.

This study presents the first sequencing and analysis of the mitochondrial genomes of *P. remota* and *F. elata elata*. Using high-throughput sequencing, we successfully obtained the mitochondrial genome sequences for both species. The mitochondrial genome of *P. remota* is 15,484 bp long (NCBI: PQ858441), and that of *F. elata elata* is 15,932 bp (NCBI: PV693697) (the non-coding region is incomplete and will not be discussed here). The mitochondrial genomes of both species comprise 37 genes (Figure 1). Gene distribution analysis revealed that 23 genes (including 14 tRNA genes and 9 PCGs) are situated in the heavy chain (H chain), whereas the remaining 14 genes (comprising 8 tRNA genes, 4 PCGs, and 2 rRNA genes) are located in the light chain (L chain). The *P. remota* genome contains 8 gene spacers and 13 gene overlaps, with the largest spacer (68 bp) found between *trnQ* and *trnM*, and the longest overlaps (8 bp) identified between the *trnW* and *trnC* genes. In contrast, the *F. elata elata* genome features 13 gene spacers and 12 gene overlap regions. Its largest spacer (37 bp) is also located between *trnQ* and *trnM*, while the longest overlapping regions (7 bp) occur between the *atp8* and *atp6* genes, as well as between the *nad4* and *nad4l* genes (Table 1). The base composition of *P. remota* is as follows: A = 39%, T = 40.07%, G = 7.98%, C = 12.96%, resulting in an AT content of 79.06% and a GC content of 20.94%. In contrast, the base composition of *F. elata elata* is A = 38.5%, T = 41.22%, G = 7.97%, C = 12.31%, yielding an AT content of 79.72% and a GC content of 20.28%. Further analysis of base bias revealed that the whole genomes of both flea species exhibited negative AT-Skew (*P. remota*: −0.01; *F. elata elata*: −0.03) and negative GC-Skew (*P. remota*: −0.24; *F. elata elata*: −0.21). These results indicate a higher proportion of the T base compared to the A base, as well as a higher proportion of the C base compared to the G base. Additionally, the contents of A + T in various functional regions demonstrated a trend of rRNAs > tRNAs > PCGs (Table 2).

**Table 1:** Mitochondrial genomic organization of *Palaeopsylla remota*/*Frontopsylla elata elata.*

Gene	Strand	Positions	Size (bp)	Initiation codon	Termination codon	Anticodon	Intergenic nucleotides
*trnI*	H	200–262/1–63	63/63			GAT	−3/26
*trnQ*	L	260–328/90–158	69/69			TTG	68/37
*trnM*	H	397–461/196–262	65/67			CAT	0/0
*nad2*	H	462–1,475/263–1,273	1014/1011	ATT/ATT	TAA/TAA		−2/−2
*trnW*	H	1,474–1,538/1,272–1,336	65/65			TCA	−8/−1
*trnC*	L	1,531–1,592/1,336–1,397	62/62			GCA	0/0
*trnY*	L	1,593–1,656/1,398–1,460	64/63			GTA	−3/−3
*cox1*	H	1,654–3,192/1,458–2,993	1539/1536	ATC/ATC	TAA/TAA		1/4
*trnL2*	H	3,194–3,257/2,998–3,061	64/64			TAA	0/1
*cox2*	H	3,258–3,933/3,063–3,743	676/681	ATT/ATG	T/TAA		0/2
*trnK*	H	3,934–4,005/3,746–3,815	72/70			CTT	−1/−1
*trnD*	H	4,005–4,067/3,815–3,879	63/65			GTC	0/0
*atp8*	H	4,068–4,226/3,880–4,050	159/171	ATT/ATT	TAA/TAA		−7/−7
*atp6*	H	4,220–4,891/4,044–4,718	672/675	ATG/ATG	TAA/TAA		−1/−1
*cox3*	H	4,891–5,673/4,718–5,500	783/783	ATG/ATG	TAA/TAA		0/0
*trnG*	H	5,674–5,735/5,501–5,563	62/63			TCC	0/−3
*nad3*	H	5,736–6,086/5,561–5,914	351/354	ATT/ATA	TAG/TAG		−2/−2
*trnA*	H	6,085–6,147/5,913–5,977	63/65			TGC	−1/−2
*trnR*	H	6,147–6,208/5,976–6,039	62/64			TCG	6/8
*trnN*	H	6,215–6,277/6,048–6,112	63/65			GTT	0/0
*trnS1*	H	6,278–6,346/6,113–6,181	69/69			TCT	0/5
*trnE*	H	6,347–6,410/6,187–6,251	64/65			TTC	−2/−2
*trnF*	L	6,409–6,470/6,250–6,314	62/65			GAA	0/0
*nad5*	L	6,471–8,187/6,315–8,048	1717/1734	ATG/ATG	T/TAA		0/1
*trnH*	L	8,188–8,251/8,050–8,115	64/66			GTG	−1/0
*nad4*	L	8,251–9,585/8,116–9,451	1335/1336	ATG/ATG	TAA/T		−7/−7
*nad4l*	L	9,579–9,872/9,445–9,738	294/294	ATG/ATG	TAA/TAA		2/2
*trnT*	H	9,875–9,937/9,741–9,805	63/65			TGT	0/0
*trnP*	L	9,938–10,000/9,806–9,868	63/63			TGG	2/2
*nad6*	H	10,003–10,515/9,871–10,383	513/513	ATA/ATT	TAA/TAA		−1/−1
*cytb*	H	10,515–11,651/10,383–11,522	1137/1140	ATG/ATG	TAA/TAA		2/3
*trnS2*	H	11,654–11,717/11,526–11,591	64/66			TGA	17/21
*nad1*	L	11,735–12,670/11,613–12,545	936/933	ATG/ATG	TAA/TAA		1/1
*trnL1*	L	12,672–12,733/12,547–12,608	62/62			TAG	0/0
*rrnL*	L	12,734–14,035/12,609–13,923	1302/1315				0/0
*trnV*	L	14,036–14,103/13,924–13,990	68/67			TAC	0/0
*rrnS*	L	14,104–14,884/13,991–14,775	781/785				0/0
control region	H	14,885–15,484/14,776–15,932	600/1157				

**Table 2:** Composition and skewness of *Palaeopsylla remota*/*Frontopsylla elata elata* mitogenome.

Region	A%	T%	G%	C%	A + T%	G + C%	AT-Skew	GC-Skew
Whole genome	39.00/38.50	40.07/41.22	7.98/7.97	12.96/12.31	79.06/79.72	20.94/20.28	−0.01/−0.03	−0.24/−0.21
PCGs	33.33/33.42	44.29/44.56	11.55/11.45	10.83/10.57	77.62/77.98	22.38/22.02	−0.14/−0.14	0.03/0.04
tRNAs	40.32/40.06	39.12/39.99	11.37/11.03	9.18/8.93	79.45/80.04	20.55/19.96	0.02/0.00	0.11/0.11
rRNAs	39.56/42.33	41.62/40.00	12.91/11.76	5.90/5.90	81.18/82.33	18.82/17.67	−0.03/0.03	0.37/0.33
*nad2*	35.21/35.31	46.55/47.77	6.80/7.02	11.44/9.89	81.76/83.09	18.24/16.91	−0.14/−0.15	−0.25/−0.17
*cox1*	29.63/29.62	41.07/41.47	13.91/14.00	15.40/14.91	70.70/71.09	29.30/28.91	−0.16/−0.17	−0.05/−0.03
*cox2*	35.21/35.24	40.83/42.29	9.91/9.84	14.05/12.63	76.04/77.53	23.96/22.47	−0.07/−0.09	−0.17/−0.12
*atp8*	37.74/40.35	46.54/46.78	3.77/4.09	11.95/8.77	84.28/87.13	15.72/12.87	−0.10/−0.07	−0.52/−0.36
*atp6*	33.04/32.74	44.05/45.04	9.52/9.19	13.39/13.04	77.08/77.78	22.92/22.22	−0.14/−0.16	−0.17/−0.17
*cox3*	31.80/30.40	42.02/42.91	12.01/11.88	14.18/14.81	73.82/73.31	26.18/26.69	−0.14/−0.17	−0.08/−0.11
*nad3*	32.19/30.79	47.58/48.31	8.26/7.06	11.97/13.84	79.77/79.10	20.23/20.90	−0.19/−0.22	−0.18/−0.32
*nad5*	35.64/36.85	44.44/43.94	13.45/12.34	6.46/6.86	80.08/80.80	19.92/19.20	−0.11/−0.09	0.35/0.29
*nad4*	32.88/34.13	45.84/44.91	13.63/13.77	7.64/7.19	78.73/79.04	21.27/20.96	−0.16/−0.14	0.28/0.31
*nad4l*	34.01/37.76	47.96/45.24	14.63/13.27	3.40/3.74	81.97/82.99	18.03/17.01	−0.17/−0.09	0.62/0.56
*nad6*	40.35/34.70	46.78/50.29	4.48/5.85	8.38/9.16	87.13/84.99	12.87/15.01	−0.07/−0.18	−0.30/−0.22
*cytb*	31.93/31.84	42.57/43.16	10.64/11.05	14.86/13.95	74.49/75.00	25.51/25.00	−0.14/−0.15	−0.17/−0.12
*nad1*	31.20/31.51	47.22/45.98	15.17/15.54	6.41/6.97	78.42/77.49	21.58/22.51	−0.20/−0.19	0.41/0.38
*rrnL*	39.40/42.81	41.78/39.92	12.90/11.79	5.91/5.48	81.18/82.74	18.82/17.26	−0.03/0.03	0.37/0.37
*rrnS*	39.82/41.53	41.36/40.13	12.93/11.72	5.89/6.62	81.18/81.66	18.82/18.34	−0.02/0.02	0.37/0.28

### 3.2 Protein-coding genes and RNA genes

Among the PCGs of *P. remota*, the longest gene is *nad5*, measuring 1,717 bp, while the shortest is *atp8*, at only 159 bp. The gene exhibiting the highest AT content is *nad6*, which reaches 87.13%. Similarly, in *F. elata elata*, the longest PCG is also *nad5*, at 1,734 bp, and the shortest is *atp8*, at 171 bp. Notably, the *atp8* gene in this species has the highest AT content, also accounting for 87.13%. Regarding nucleotide skewness, the AT-Skew is negative for all 13 PCGs in both species. The GC-Skew is predominantly negative, except for *nad5*, *nad4*, *nad4l*, and *nad1*, which show positive GC-Skews in both *P. remota* and *F. elata elata*. All 13 PCGs in both species use ATN as the start codon. For stop codons, most genes terminate with TAA. Exceptions are observed in *P. remota*, where *nad3* uses TAG, and *nad5* and *cox2* use an incomplete stop codon T. In *F. elata elata*, *nad3* uses TAG, and *nad4* uses an incomplete stop codon T (Tables 1, 2).

**Figure 2:**
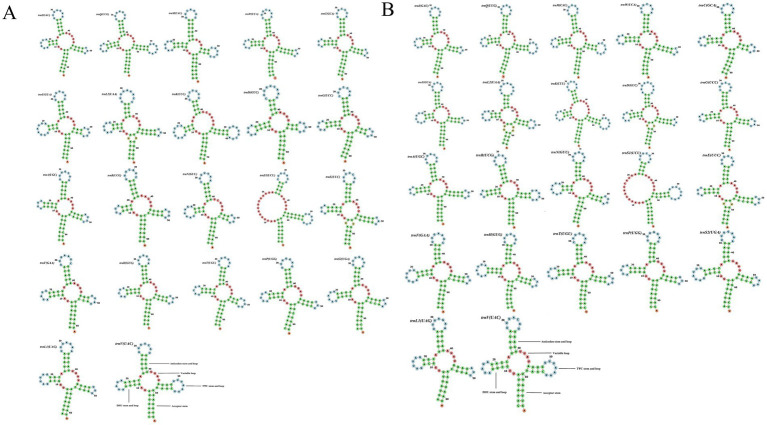
Cloverleaf structures of the 22 tRNA genes from *Palaeopsylla remota* **(A)** and *Frontopsylla elata elata* **(B)**.

The lengths of the 22 tRNA genes of *P. remota* range from 62 to 72 bp, with the *trnK* gene (72 bp) being the longest. In contrast, the 22 tRNA genes of *F. elata elata* range from 62 to 70 bp, with its *trnK* gene (70 bp) also being the longest. In the mitochondrial genomes of both species, all tRNA genes, except for *trnS1 (TCT)*, which lacks the DHU arm, exhibit typical cloverleaf secondary structures. During the formation of the secondary structures of tRNA genes, most bases adhere to the principle of complementary base pairing; however, instances of base mismatches do occur. Analysis revealed a total of 19 mismatches (17 G-U mismatches and 2 U–U mismatches) in the tRNAs of *P. remota*, while *F. elata elata* exhibited 21 mismatches (17 G-U mismatches and 4 U–U mismatches) (Figure 2). Furthermore, ribosomal RNA gene analysis indicated that the lengths of *rrnL* and *rrnS* in *P. remota* are 1,302 bp and 781 bp, respectively, whereas the corresponding gene lengths in *F. elata elata* are 1,315 bp and 785 bp, respectively. In both species, the *rrnL* and *rrnS* genes are separated by the *trnV* gene.

### 3.3 Codon preference analysis of *Palaeopsylla remota* and *Frontopsylla elata elata*

An RSCU value of 1 indicates no bias in codon usage. When RSCU is greater than 1, it suggests that the codon is used more frequently than expected, whereas an RSCU value less than 1 indicates lower-than-expected usage frequency. Specifically, an RSCU value exceeding 1.6 is considered indicative of overexpression, while a value below 0.6 suggests underexpression (34, 44–46). The RSCU analysis in this study revealed that *P. remota* possesses 13 overexpressed codons and 20 underexpressed codons. Among the overexpressed codons, UUA (3.01), CCU (2.28), GCU (2.16), and GGA (2.00) exhibited the highest RSCU values (Supplementary Figure S2A1). In contrast, *F. elata elata* has 19 overexpressed codons and 31 underexpressed codons, with UUA (4.66), GCU (2.51), CGA (2.36), GUU (2.18), and GGA (2.15) showing the highest RSCU values (Supplementary Figure S2B1).

The ENC is a critical indicator used to assess the extent of unequal preference in synonymous codon usage, with a theoretical value ranging from 20 to 61. A low ENC value (ENC < 35) indicates a strong codon usage bias, whereas a higher ENC value suggests a weaker or negligible preference (46). In the case of *P. remota*, the ENC values of its 13 PCGs range from 24.6 to 34.48, indicating a significant codon usage bias across all these genes. Furthermore, all 13 genes are located below the standard curve, suggesting that their codon usage patterns are primarily shaped by natural selection (Supplementary Figure S2A2). For *F. elata elata*, the ENC values of 12 PCGs range from 25.81 to 40.59 (The *atp8* gene was excluded from the analysis because it does not contain any amino acids encoded by fourfold degenerate synonymous codons, making it impossible to calculate its ENC value.). The results indicate that, except for the *nad4l* gene (ENC = 40.59), the remaining 11 PCGs exhibit a clear codon usage bias. Moreover, all these 11 genes are located below the standard curve, further supporting the conclusion that natural selection is the primary driving force behind their codon usage preferences (Supplementary Figure S2B2).

PR2 analysis revealed that the majority of mitochondrial PCGs in *P. remota* and *F. elata elata* were situated in the first quadrant (A3/(A3 + T3) > 0.5, G3/(G3 + C3) > 0.5) and the second quadrant (A3/(A3 + T3) > 0.5, G3/(G3 + C3) < 0.5). Specifically, three genes of *P. remota* were identified in the first quadrant, while eight genes were found in the second quadrant (Supplementary Figure S2A3). In contrast, for *F. elata elata*, four genes were located in the first quadrant, seven genes in the second quadrant, and one gene in the third quadrant (Supplementary Figure S2B3). Genes in the first quadrant exhibit higher A than U and higher G than C usage at the third codon position. Those in the second quadrant show A > U but C > G, while the third quadrant displays U > A and C > G (47).

### 3.4 Analysis of protein-coding genes in the mitochondrial genome of Siphonaptera

**Figure 3:**
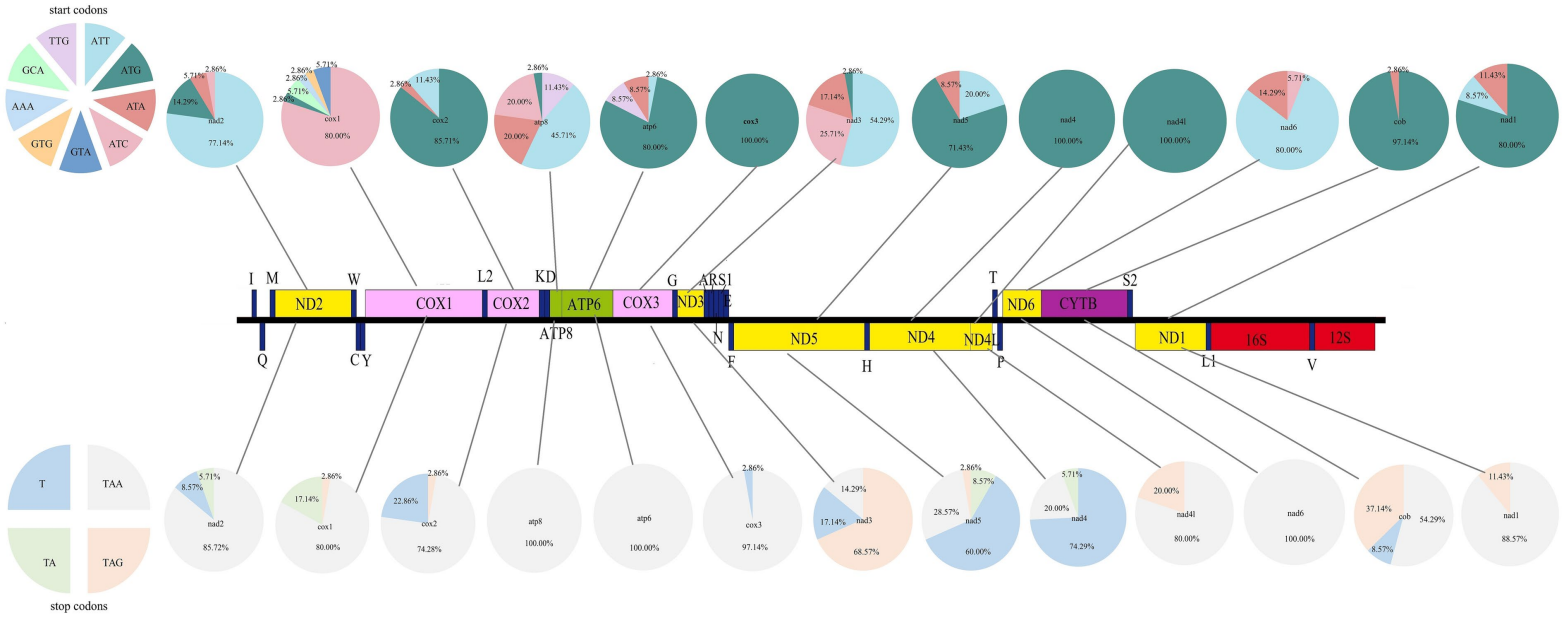
Analysis of start and stop codon usage in Siphonaptera mitogenomes. Pie charts characterizing the frequency distribution for the 13 PCGs.

Analysis of the mitochondrial genomes of fleas (available in the NCBI database) (Table 3) indicates that among the 13 PCGs, the majority utilize the canonical ATN start codon. Specifically, the genes *cox2*, *atp6*, *cox3*, *nad5*, *nad4*, *nad4l*, *cytb*, and *nad1* predominantly employ ATG as the start codon. The genes *nad2*, *nad3*, and *nad6* more frequently use ATT, while *cox1* primarily initiates with ATC. Additionally, several non-standard start codons have been observed. For instance, in *cox1*, the start codons GTA, CTG, GCA, and AAA were identified, whereas TTG was found in *atp8* and *atp6*. Regarding stop codons, TAA is the most commonly used among the 13 genes. However, *nad5* and *nad4* frequently utilize the incomplete stop codon T, and TAG is predominantly used by *nad3*, distinguishing them from the other genes (Figure 3).

**Table 3:** Species, GenBank accession number and length of mitogenomes used in this study.

Superfamily	Family	Species	Size (bp)	GenBank accession number
Pulicoidea	Pulicidae	*Ctenocephalides orientis*	22,189 bp	NC_073009
		*Pulex irritans*	20,337 bp	NC_063709
		*Ctenocephalides canis*	15,609 bp	NC_063710
		*Ctenocephalides felis*	20,873 bp	NC_049858
		*Xenopsylla cheopis*	18,902 bp	MW310242
		*Ctenocephalides felis felis*	20,911 bp	MW420044
	Tungidae	*Tunga penetrans*	17,279 bp	PV426769
Vermipsylloidea	Vermipsyllidae	*Dorcadia ioffi*	16,785 bp	NC_036066
Pygiopsylloidea	Stivaliidae	*Aviostivalius klossi bispiniformis*	18,669 bp	PP963728
Hystrichopsylloidea	Hystrichopsyllidae	*Hystrichopsylla weida qinlingensis*	17,173 bp	NC_042380
	Ctenophthalmidae	*Stenoponia polyspina*	14,933 bp	OR834393
		*Ctenophthalmus yunnanus*	15,801 bp	NC_085277
		*Stenischia montanis yunlongensis*	15,651 bp	OR780663
		*Stenischia montanis*	15,889 bp	PP990561
		*Stenischia humilis*	15,617 bp	NC_073020
		*Neopsylla hongyangensis*	15,832 bp	PP133648
		*Ctenophthalmus quadratus*	15,938 bp	NC_072692
		*Palaeopsylla remota*	15,484 bp	PQ858441
		*Neopsylla specialis*	16,820 bp	NC_073019
Ceratophylloidea	Ischnopsyllidae	*Thaumapsylla breviceps orientalis*	15,631 bp	PP973737
	Leptopsyllidae	*Frontopsylla diqingensis*	16,153 bp	PP083946
		*Paradoxopsyllus custodis*	15,375 bp	OQ627398
		*Frontopsylla spadix*	15,085 bp	NC_073018
		*Leptopsylla segnis*	15,785 bp	NC_072691
		*Frontopsylla elata elata*	15,932 bp	PV693697
		*Amphipsylla qinghaiensis*	15,579 bp	PQ571081
	Ceratophyllidae	*Ceratophyllus wui*	18,081 bp	NC_040301
		*Jellisonia amadoi*	17,031 bp	NC_022710
		*Amphalius spirataenius*	14,825 bp	OR855715
		*Nosopsyllus laeviceps*	16,533 bp	PP838812
		*Citellophilus tesquorum*	15,345 bp	NC_088096
		*Macrostylophora euteles*	16,027 bp	NC_085274
		*Ceratophyllus anisus*	15,875 bp	NC_073017
		*Citellophilus tesquorum mongolicus*	15,373 bp	PV693696
		*Citellophilus tesquorum dzetysuensis*	16,458 bp	PV693698

**Figure 4:**
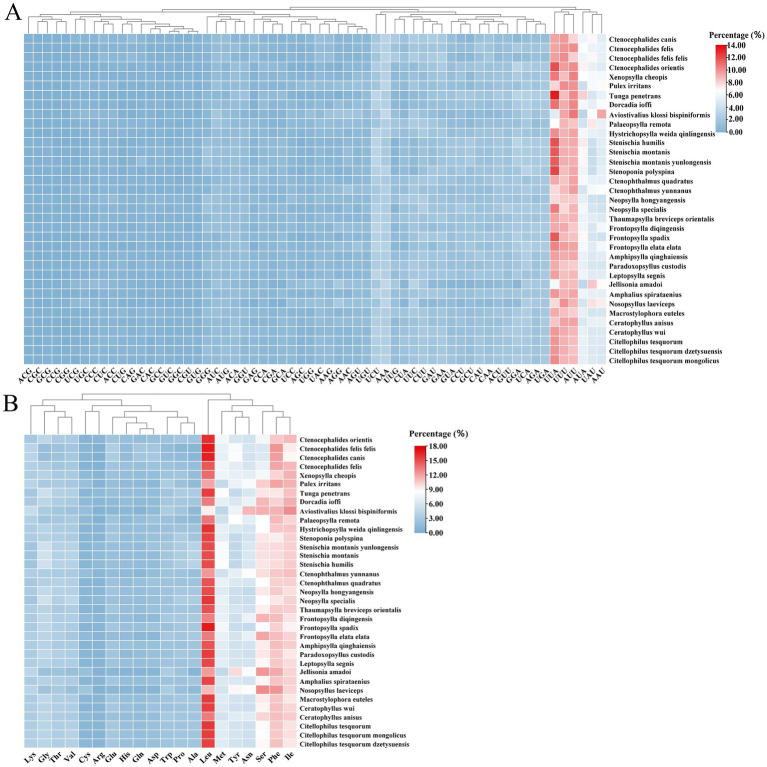
Codon and amino acid usage frequency heatmap for mitochondrial protein-coding genes in Siphonaptera. **(A)** Codon usage frequency; **(B)** Amino acid usage frequency.

The usage patterns of codons and amino acids in 13 PCGs of Siphonaptera were examined. The analysis revealed that the codons exhibiting the highest frequency of usage were UUA, UUU, and AUU. In terms of amino acid frequency, leucine (Leu) emerged as the most prevalent, followed closely by phenylalanine (Phe), isoleucine (Ile), and serine (Ser) (Figure 4).

### 3.5 Non-synonymous/synonymous substitution ratio and sliding window analysis

**Figure 5:**
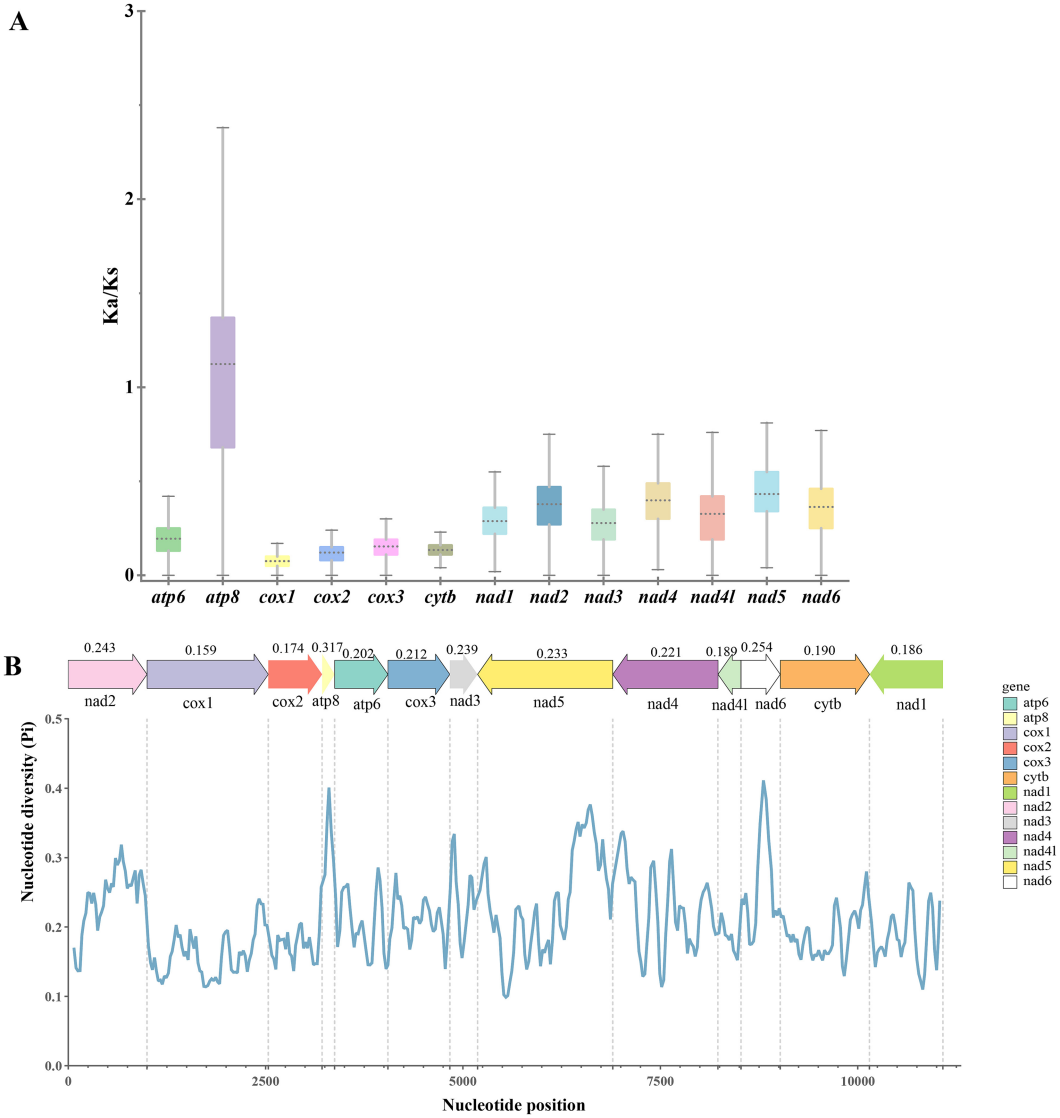
Non-synonymous (Ka) and synonymous (Ks) substitution rates **(A)** and nucleotide diversity (pi) **(B)** in Siphonaptera mitochondrial genomes.

It is generally accepted that a Ka/Ks ratio >1 implies positive selection, ≈1 refers to neutral evolution, and <1 indicates purifying selection (48). The estimated Ka/Ks ratios of 13 PCGs within the order Siphonaptera were calculated, and the results showed that the ratios were arranged in descending order as follows: *atp8* > *nad5* > *nad4* > *nad2* > *nad6* > *nad4l* > *nad1* > *nad3* > *atp6* > *cox3* > *cytb* > *cox2* > *cox1*. Only the *atp8* gene showed a Ka/Ks ratio greater than 1, indicating that it is under positive selection and evolving at a relatively faster rate. The other genes had Ka/Ks ratios all below 1 suggesting they are under purifying selection. The *cox1* gene exhibited the lowest Ka/Ks value, indicating it is under the strongest purifying selection, has the slowest evolutionary rate, and is the most conserved gene among all analyzed genes (Figure 5A).

In addition, the nucleotide diversity (Pi) of the 13 PCGs in the order Siphonaptera ranged from 0.10 to 0.45. Sliding window analysis revealed that the *atp8* gene had the highest nucleotide diversity (average Pi = 0.317), followed by the *nad6* and *nad2* genes. In contrast, the *cox1* gene showed the lowest nucleotide diversity (average Pi = 0.159) (Figure 5B).

### 3.6 Phylogenetic analysis

**Figure 6:**
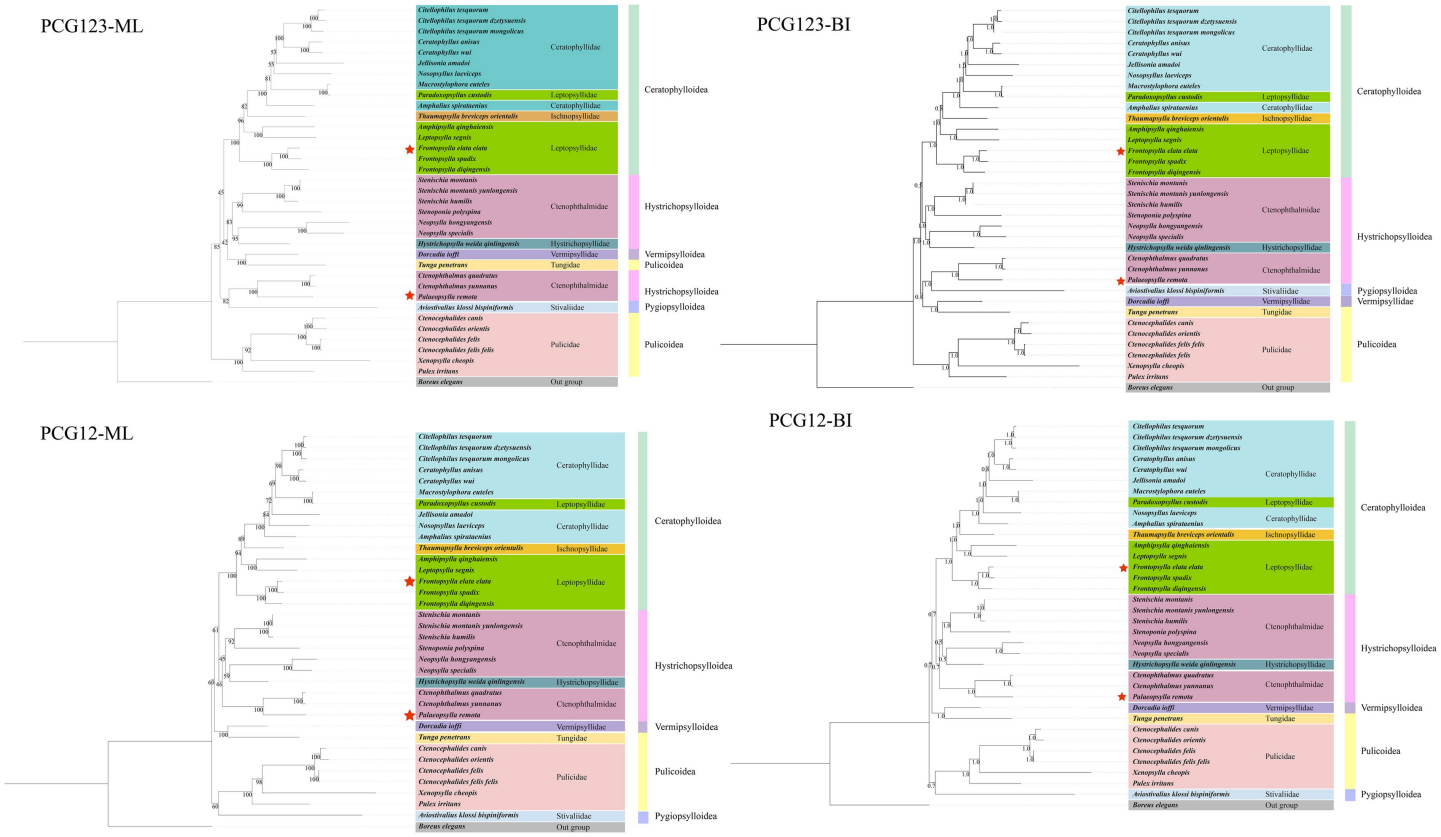
Phylogenetic reconstruction of Siphonaptera based on PCG123 and PCG12 datasets using Bayesian inference and maximum likelihood.

Using the PCG123 and PCG12 datasets, four phylogenetic trees were created employing the ML and BI approaches. The analysis results indicate that in all trees, species of the order Siphonaptera are divided into two major lineages, although their composition varies across different data matrices. In the PCG123 matrix, the first branch includes Tungidae, Vermipsyllidae, Stivaliidae, Hystrichopsyllidae, Ctenophthalmidae, Ischnopsyllidae, Leptopsyllidae, and Ceratophyllidae. The second branch contains only Pulicidae. In the PCG12 matrix, the first branch includes Tungidae, Vermipsyllidae, Hystrichopsyllidae, Ctenophthalmidae, Ischnopsyllidae, Leptopsyllidae, and Ceratophyllidae, while the second branch comprises Pulicidae and Stivaliidae. All four trees unanimously support the monophyly of the Pulicidae family and the Ceratophylloidea superfamily, whereas the Ceratophyllidae, Leptopsyllidae, and Ctenophthalmidae families are found to be paraphyletic. Furthermore, in all topological structures, *P. remota* forms a sister group with the clade (*Ctenophthalmus quadratus* + *C. yunnanus*) (BS = 100, PP = 1), indicating a relatively close phylogenetic relationship. Meanwhile, *F. elata elata* is identified as the sister taxon to the clade ((*F. elata elata* + *F. spadix*) + *F. diqingensis*) (BS = 100, PP = 1) (Figure 6).

### 3.7 Divergence time estimation

**Figure 7:**
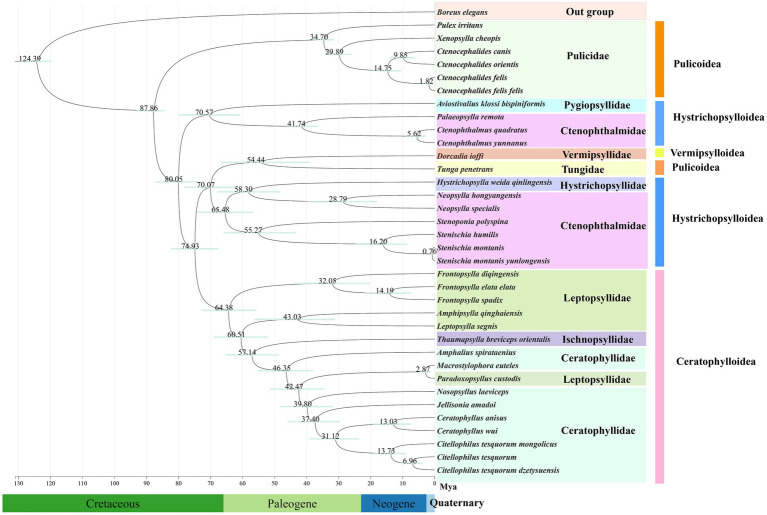
Estimated divergence times within Siphonaptera. Node labels show mean ages; bars represent 95% confidence intervals.

Based on divergence time estimates, the species of Siphonaptera examined in this study originated during the Cretaceous period, approximately 124.39 Mya. Subsequently, the major lineages within this order underwent progressive differentiation: Clade I (Pulicidae) diverged from Clade II (comprising Pygiopsyllidae, Vermipsyllidae, Tungidae, Hystrichopsyllidae, Ctenophthalmidae, Ischnopsyllidae, Leptopsyllidae, and Ceratophyllidae) around 87.86 Mya. Within Clade II, divergence times varied among families: the earliest divergence of Ctenophthalmidae occurred at approximately 70.57 Mya, Leptopsyllidae at about 64.38 Mya, and Ceratophyllidae at around 57.14 Mya. In addition, the superfamily Ceratophylloidea diverged relatively early, at approximately 74.93 Mya. It is noteworthy that although the ancestral lineages of fleas emerged as early as the Cretaceous, molecular clock analyses indicate that substantial diversification events in most extant lineages primarily occurred after the Cretaceous-Paleogene (K-Pg) boundary (Figure 7).

## 4 Discussion

This study presents the inaugural analysis of the mitochondrial genomes of *P. remota* and *F. elata elata*. Both flea species possess mitochondrial genomes that comprise the standard 37 genes along with one control region. An analysis of nucleotide composition revealed a pronounced A + T bias in both flea species. A high A + T content is a common feature of insect mitochondrial genomes, as demonstrated in multiple studies. For example, Wei et al. analyzed the base composition of 120 insect mitochondrial genomes and revealed that the relative A + T content is affected by gene direction, replication processes, and codon position (49). Previous studies have also indicated that a parasitic lifestyle can accelerate mitochondrial mutation rates, leading to an increase in AT content (50). Among the 22 tRNAs in the two flea species, only *trnS1* lacks the DHU arm. This absence of the DHU arm has been documented in insects, mammals, and other metazoan lineages. Garey and Wolstenholme proposed that the loss of the DHU arm in *trnS1* occurred early in metazoan evolution (51, 52). Furthermore, tRNAs from both species contain G-U mismatches. These non-canonical base pairs can enhance the structural stability of tRNAs, a phenomenon widely observed across various groups, including insects and mammals. Studies have shown that G-U mismatches can serve as an intermediate bridge in compensatory evolution, enabling the evolutionary process to more easily cross fitness valley (53, 54). Analysis of start and stop codon usage in the 13 PCGs of the studied flea species showed that most genes employ the conventional ATN start codon. However, non-canonical start codons, such as GTA, CTG, GCA, and AAA, were identified in *cox1*. Previous studies have documented the presence of non-canonical start codons in *cox1* across various insect groups, including Diptera, Lepidoptera, Orthoptera, and Coleoptera (52, 55–57). One hypothesis posits that the adoption of non-canonical start codons in *cox1* may serve as an optimization strategy aimed at maximizing coding density while minimizing transcriptional conflicts (52). Furthermore, incomplete stop codons (T and TA) were detected in multiple PCGs. Such incomplete stop codons are prevalent among metazoans and can be completed through post-transcriptional polyadenylation, which adds A or AA to form the complete stop codon TAA (53, 58, 59).

The genetic code’s degeneracy allows numerous amino acids to be represented by several synonymous codons, and various organisms exhibit differences in their spontaneous codon usage frequency. These variations in codon selection could also affect protein expression, structure, and function (60). Through the examination of the relative synonymous codon usage (RSCU) among 13 PCGs in *P. remota* and *F. elata elata*, we evaluated the preferences for synonymous codon utilization. An RSCU value of 1.0 suggests an absence of preference; values surpassing 1.0 indicate a usage of codons that is greater than anticipated, whereas values falling below 1.0 signify a usage that is less than expected. We found that the majority of codons terminating in A/U displayed RSCU values above 1. Previous studies have shown that species with high AT content exhibit a stronger preference for A/U ending codons, a common phenomenon in metazoan mitochondria (61, 62). ENC-plot analysis indicated that all 13 PCGs of *P. remota* and 11 PCGs of *F. elata elata* lie below the standard curve, suggesting that their codon usage preferences are influenced by natural selection. Additionally, this research conducted an analysis of selection pressure on 13 PCGs from Siphonaptera species. The results showed that, with the exception of the *atp8* gene, the Ka/Ks ratios for the other 12 PCGs were all below 1. This suggests that the *atp8* gene has experienced positive selection, whereas the *cox1* gene exhibited the lowest Ka/Ks ratio, indicative of the strongest purifying selection pressure and thus a suitable candidate for DNA barcoding. Furthermore, this pattern of purifying selection is consistent with those reported in other hematophagous ectoparasites, including mosquitoes, ticks, and mites (63–66). Additionally, nucleotide diversity (Pi) analysis revealed that the *cox1* gene demonstrates the highest conservation, further confirming its suitability as a molecular marker for species identification. Conversely, the *atp8* gene, which possesses higher Pi values, is more appropriate for studies on species evolution (67).

Whiting et al. proposed that fleas primarily parasitizing Australian marsupials and monotremes, including families such as Pygiopsyllidae, Macropsyllidae, and Stephanocyllidae, represent an early-branching lineage. In the ML and BI trees constructed from the PCG12 dataset in this study, Stivaliidae (originally described as Pygiopsyllidae) consistently occupied basal positions within the phylogenetic tree, aligning with this hypothesis. Furthermore, our findings corroborate another inference by Whiting et al. that fleas most prevalent and diverse in northern temperate regions, represented by the superfamily Ceratophylloidea, form a more recent clade. This is further supported by the placement of Ceratophylloidea within the most derived clades of all four phylogenetic trees in this study (68). Additionally, this study supports the monophyly of the superfamily Ceratophylloidea, whereas both Hystrichopsylloidea and Pulicoidea were recovered as paraphyletic. This finding is highly similar to the proposal by Zhu et al. that Pulicomorpha and Hystrichopsyllomorpha constitute paraphyletic groups (43). Divergence time estimation indicated that the common ancestor of Siphonaptera originated in the Cretaceous, whereas divergence among its major extant lineages primarily took place after the K-Pg boundary, which aligns with the findings of Zhu et al. (43). However, because this study did not cover all taxa within Siphonaptera, there remains a certain degree of uncertainty in the currently constructed phylogenetic relationships and the estimated divergence times. Therefore, future studies should integrate additional mitochondrial genome data from all families of Siphonaptera. Such efforts are expected to more clearly resolve the evolutionary history and diversification patterns of this group, thereby enhancing the credibility of inferences and the robustness of results.

## 5 Conclusion

This study presents the first mitochondrial genome analysis of *P. remota* and *F. elata elata*. By integrating these new data with existing flea mitochondrial genomes, we systematically investigate the evolutionary patterns and taxonomic status of fleas. Phylogenetic trees constructed from both PCG123 and PCG12 datasets indicate that the superfamily Ceratophylloidea and the family Pulicidae are monophyletic. In contrast, the superfamilies Hystrichopsylloidea and Pulicoidea, as well as the families Ceratophyllidae, Leptopsyllidae, and Ctenophthalmidae, were all reconstructed as paraphyletic groups. Furthermore, Ceratophylloidea occupies the most derived position in the phylogenetic tree, suggesting it represents a relatively recent evolutionary group within the Siphonaptera order. The estimation of divergence times additionally indicates that the latest common ancestor of Siphonaptera emerged during the Cretaceous period, whereas the diversification of current lineages mainly took place following the K-Pg boundary. By incorporating molecular data from newly examined flea species, this study provides novel insights and a strengthened scientific foundation for further elucidating the evolutionary history of Siphonaptera and refining its taxonomic system.

## Data Availability

The nucleotide sequences of the *P. remota* and *F. elata elata* mitogenome were deposited in NCBI (https://www.ncbi.nlm.nih.gov/) under accession number PQ858441 and PV693697, respectively.

## Footnotes

1
https://github.com/OpenGene/fastp

2
https://github.com/Kinggerm/GetOrganelle

3
https://github.com/rrwick/Bandage

4
http://mitos.bioinf.uni-leipzig.de/

5
https://itol.embl.de/itol.cgi
