# Supplementary material for: Mitogenomic analysis of Palaeopsylla remota and Frontopsylla elata elata with implications for the phylogeny of Siphonaptera
Source: Front Vet Sci. 2026 May 12;13:1722404. doi: 10.3389/fvets.2026.1722404 (PMC13201117; doi:10.3389/fvets.2026.1722404)
Supplement: Supplementary file 1 [file Supplementary_file_1.doc]

***Supplementary Material***

**Supplementary Figures**

**
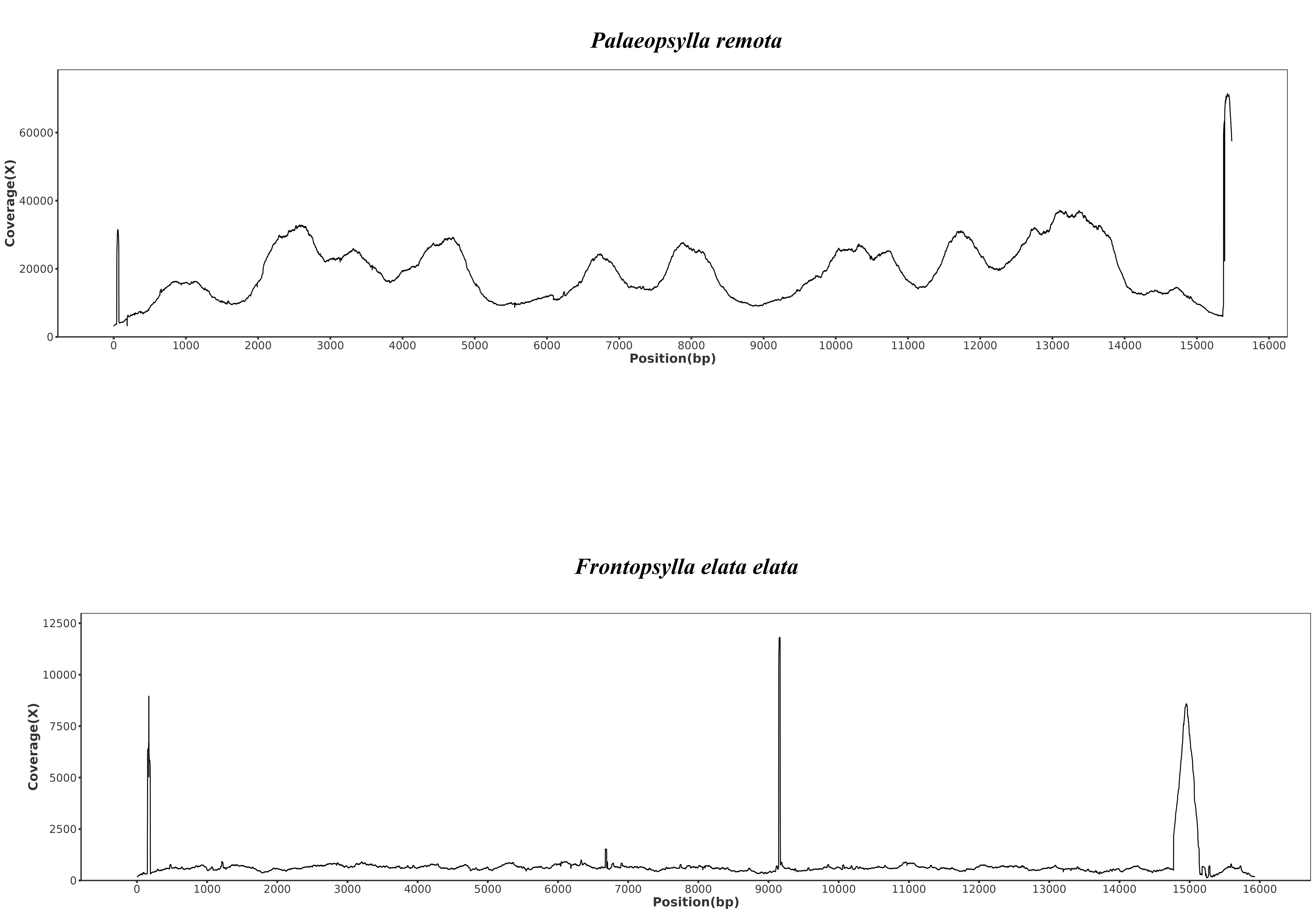
**

Figure S1 Sequencing depth plots of *Palaeopsylla remota* and *Frontopsylla elata elata*

**

**

Figure S2 Codon usage preference analysis in *Palaeopsylla remot*a and *Frontopsylla elata elata*. (A) RSCU plot, (B) ENC-plot, (C) PR2 plot
